# What PIs want when hiring a clinical research coordinator

**DOI:** 10.1017/cts.2024.505

**Published:** 2024-04-15

**Authors:** Elaine Fisher, Rebecca Thomas, Ikseon Choi, Linda McCauley

**Affiliations:** 1 Emory University, Nell Hodgson Woodruff School of Nursing, Atlanta, GA, USA; 2 CenExel: Medical Research Centers, Atlanta, GA, USA

**Keywords:** Clinical research coordinator, career navigation, human resources, training & support, workforce

## Abstract

**Introduction:**

Clinical research coordinators (CRCs) play a key role in supporting the translational research enterprise, with responsibilities encompassing tasks related to the design, implementation, and evaluation of clinical research trials. While the literature explores CRC competencies, job satisfaction, and retention, little attention has been given to the role of the PI working with Human Resources (HR) in the CRC hiring and onboarding processes. We investigated the priorities, decision-making processes, and satisfaction levels of principal investigators (PIs) and hiring managers in CRC hiring.

**Methods:**

An online survey consisting of open-ended and fixed-choice questions to gather information on desired CRC qualifications and competencies, factors influencing hiring decisions, and overall satisfaction with selected candidates was administered. The survey utilized a Task/Competency Checklist developed from job descriptions and the literature. Respondents were asked to rank the importance of factors such as CRC skill set, years of experience, educational background, and budget constraints.

**Results:**

Results indicated that the skill set of the applicant was the most frequently cited factor influencing the hiring decision, followed by years of experience. Education and budget constraints were of lesser importance. Most respondents reported a satisfaction rating of 50% or greater with their new hires, although some participants expressed challenges related to institutional training requirements, the performance of entry-level CRCs, and the qualifications of experienced candidates.

**Conclusion:**

The hiring cycle involves HR-PI collaboration for a clear job description, effective onboarding processes, and accessible professional development opportunities to enhance PI and employee satisfaction and CRC retention.

## Introduction

Clinical research coordinators (CRCs) provide a vital role in supporting the translational research enterprise. These responsibilities encompass various tasks related to study design, implementation, and evaluation of clinical research trials [1]. Research studies require CRCs to have a diverse skill set including the ability to recruit participants, manage sample and inventory, document accurately, monitor for adverse events, serve as the liaison between principal investigators (PIs) and sponsors, perform data entry and management, and provide direct care and follow-up, to name a few [2–5]. While the literature is rapidly expanding on the extent to which CRCs are competent to perform their jobs and attributes contributing to job satisfaction and retention, little attention has been given to the role of Human Resources (HR) in hiring and onboarding individuals into these positions. Hiring a CRC involves a substantial investment of time and training to ensure proper job performance [2]. Making an incorrect hiring decision can be costly not only in lost salary but also lost time in study initiation and for retraining an individual to fill the role [6].

For some PIs, hiring a CRC represents their first staff hire after receiving initial research funding. A lack of clarity on the role the CRC will play in implementing the research project leads to confusion over job responsibilities often resulting in the PI defaulting to a generic institutional job description. In conversations with hiring managers and PIs, it was common to hear expressions like, “I’ll know the right candidate when I see them” and frustration with HR’s understanding of their position requirements resulting in the submission of unsuitable candidate resumes for consideration.

The initial step in hiring a CRC involves collaborating with HR to identify project-specific needs prior to posting the position. In larger institutions, HR recruiters play a critical role in editing and posting the position and conducting initial screenings of applicants to assess the fit between applicants’ qualifications and job requirements. We have developed a career navigation system to assist CRCs and other research professionals to understand their competencies, and areas in which their performance can be enhanced with training [7]. As we developed the system, we communicated with HR recruiters in our system to learn more about their perceptions of our navigation system and the challenges they face in hiring the right individuals for research projects. They expressed an ongoing challenge of effectively screening a diverse applicant pool and aligning candidate resumes with the study needs identified by PIs. They also described the challenge of matching the CRC’s level of experience with the project requirements (Personal communication with HR recruiters and HR administrators). Research institutions often classify CRC by skill level (typically 3–4 levels) ranging from candidates having no or limited experience to advanced expertise in clinical research. In our system, CRC levels 1 & 2 represent entry level to advanced beginner positions, involving candidates with transferrable skills or up to a year of clinical research experience. The intermediate- level is CRC 3, while CRC 4 denotes an advanced level. While most institutions have developed standard position descriptions for CRCs, these descriptions may lack any specific project requirements or differentiation of required skill levels. When individuals are hired into positions at a level that exceeds or does not match their abilities, job dissatisfaction and PI disappointment ensue. To enhance the efficiency and alignment of CRC applicant abilities with posted positions, we conducted a study involving PIs and/or their hiring designees who advertised for and/or hired CRCs between 2020 and 2021. The survey aim was to explore their priorities for hiring a CRC, factors influencing their decision-making to hire, overall satisfaction with the selected candidate, and employee training needs and availability of resources post-hire.

## Materials and methods

An online survey comprised of open-ended and fixed-choice questions was used to obtain specific information on desired qualifications of CRC candidates, factors influencing their hiring decision, PI satisfaction with the hire, and training needs post-hire. Survey Monkey was used as the platform for survey creation, data collection, and result analysis.

To identify potential participants, a list of PIs or their hiring managers who had posted a CRC position(s) within a 12-month period was obtained from HR. Email invitations to participate in the online survey were sent to the identified individuals, with follow-up reminders sent to nonresponders at 1 and 3 weeks. Responders were provided an electronic survey link to complete the questionnaire. This survey received Institutional Review Board (IRB)-exempt status.

Survey components included a 32 Tasks/Competency Checklist for CRCs (Table 1), factors influencing the hiring decision, an overall satisfaction rating for the hire, and training needs. Open comment boxes were provided within the survey. The Task/Competency Checklist was developed using CRC job descriptions and from multiple sources in the literature [8–10]. To identify key tasks/competencies, a comprehensive list of 250+ tasks was compiled. Each task was placed on an index card and index cards were sorted by two (2) subject matter experts into major categories with overlapping tasks/competencies combined. This resulted in a final checklist of 32 activities performed by CRCs.

**Table 1.** Tasks/competency checklist

Prepares & tracks regulatory documents (i.e., 1572, CVs, financial disclosure forms)
Works with Institutional Review Board (IRB)/Institutional Animal Care and Use Committee (IACUC) and other regulatory committees
Prepares/submits/modifies study protocols
Administrative activities (i.e., record keeping, coordinating services, maintains equipment and supplies)
Manages study startup
Develops & maintains other study related documents
Collects & enters case report forms (ECRFs)
Processes & ships study specimens
Phlebotomy
Works with marginalized populations (i.e., children, pregnant women, persons with impaired mental status, etc.)
Screens, recruits, consents, & educates study participants
Trains/ educates research staff and other team members
Communicates with sponsors and key business partners
Develops recruitment strategies
Recognizes and manages adverse events
Data monitoring & security
Data analysis & interpretation
Manages internal study audits & inspections
Investigational product use
Pharmacovigilance
Manages large research projects
Manages multisite research projects
Leads team meetings
Manages teams
Supervisory duties (i.e., resolves study issues, identifies and implements corrective actions and processes, etc.)
Manages study budgets
Manages Pre- and Post-grant awards
Manages study close-out
Compliance monitoring
Develops presentations
Contributes to/ writes for publication
Grant writing and submission

Using the Task/Competency Checklist, respondents were asked to check all tasks/competencies important to CRC job responsibilities. Next, using the same Task/Competency Checklist they were asked to select the top five (5) tasks/competencies needed for their most recent CRC hire. Respondents were asked to rank order factors influencing their hiring decision- budget, skill set, educational background, and years of experience in clinical research; and to identify the CRC level required for their project. Within our system there are four (4) CRC levels, CRC I & II - entry level, CRC III - intermediate level, and CRC IV - advanced level. To determine satisfaction with the hire, two questions were included: (1) How satisfied were you with your last CRC hire to accomplish the work on the research project? and (2) Would you hire this individual again? The final open-ended question focused on training needs of the new hire.

## Results

We received a 38% response rate from a mix of both PI and/or their hiring managers (100 surveyed/ 38 respondents). The survey contained both open-ended and fixed-choice questions. Table 1, Frequently Identified CRC Tasks/Competencies, displays the top 5 job responsibilities selected for a CRC position hire. Thinking back to their last hire, 89% of jobs posted were for an entry-level CRC position (CRC I & CRC II), with 70% for a CRC II position. Within our institution, a CRC II position requires one year of clinical research experience. Respondents selected for their specific hire (last hire) the same 5 tasks/competencies they identified as essential for any CRC hire, regardless of the level of CRC they were hiring – entry level to advanced level (Table 2).

**Table 2.** Frequently identified clinical research coordinator (CRC) tasks/competencies

CRC Tasks/Competencies	*N* = 38	100%
Administrative activities	36	94.7%
Screening, recruiting, consenting, & educating study participants	34	89.5%
Developing & maintaining study related documents	34	89.5%
Collecting & entering case report forms	32	84.2%
Recognizing & managing adverse events	25	65.8%

Participants were asked to rank the factors most important to their last hiring decision. The skill set of the applicant was the most frequently cited factor, with 73% of participants ranking it as their top priority. This was followed by years of experience which was ranked as the second most important factor by 46% of participants. Educational level ranked third, with 51% of participants considering it significant, while the budget available for the project was ranked fourth, with 78% of participants considering it important but less so than the other factors.

Most respondents (78%) reported a satisfaction rating of 50% or greater with their new hire. One respondent mentioned that the hired individual had previous experience with the team in a different role, which gave them confidence in their fit for the position and allowed for quick training on the missing job aspects. Among those who reported a less favorable hiring experience (satisfaction rating below 50%), one participant expressed frustration with the lengthy and demanding internal institutional training requirements, which took up to 6 months to complete. Another participant stated that an entry-level CRC was unable to perform the expected tasks for the project, while others mentioned that experienced candidates turned out to be less qualified than anticipated. Commonly missing skills reported included phlebotomy, knowledge of institutional policies and procedures, a deep understanding of specific study protocol elements, and a lack of experience with human participants/IRB. Sixteen percent (16%) of participants stated they would not hire the individual again.

## Discussion

The CRC hiring process involves creating a comprehensive job description based on research project requirements and clear communication with an HR recruiter during the applicant review process, onboarding, and post-hire for training and retention. The CRC Tasks/Competencies Checklist includes general day-to-day operations tasks and specialized tasks like data analysis and reporting and writing for publication/grants, suitable for experienced candidates. Respondents identified the same five top tasks for their recent CRC hire, all focusing on day-to-day operations. The lack of distinguishing qualifications between CRC job levels poses a challenge for HR recruiters in matching candidates to PI and project needs and contributes to varied levels of satisfaction with the employee post-hire. To promote great CRC hires that match the PI expectations and the CRCs' skill set, new approaches are needed in three major areas: (1) Analysis of the skill set of CRCs seeking employment opportunities, (2) Professional trainings that are accessible to CRCs, low or no cost, and allow the individual to build new skills, and (3) Engagement of PIs with HR in structuring job descriptions that match the project needs and describe accurately the skills that are needed.

### Skill set analysis

Skill set was the top consideration when hiring a CRC. The responsibility lies with the PI or the designated hiring manager to communicate this in the job description and to the HR recruiter reviewing candidate applications. New PIs with limited familiarity rely on HR recruiters to understand qualifications, leading to potential issues in cases when CRCs are asked to take on responsibilities beyond their scope, risking both the PI and the institution [11,12]. Specific job descriptions are essential for matching candidates effectively, considering the diverse research foci and budget constraints. Hiring entry-level CRCs without proper qualifications to meet job requirements negatively affects job satisfaction and retention [13].

HR departments bear a responsibility of understanding the skill levels of CRCs, as it plays a crucial role in predicting retention and job satisfaction of CRCs [2]. CRCs are expected to possess additional skills, training, and medical knowledge [12], including knowledge of disease processes, research regulations, participant management, health information privacy laws, policies, procedures, Good Clinical Practice, and site- and study-specific knowledge, either explicitly or implicitly. Additionally, to address diversity among CRCs and implement effective strategies for working with specific populations, addressing disparities in people from underrepresented backgrounds requires training in recruitment strategies and cultural humility. This training should consider the impact of social determinants of health [14].

### Promoting skill development and career navigation

Solutions are needed to assist organizations and individual PIs in hiring the appropriate skill set for their projects. The eMPACT^TM^ system was designed with funding from the Georgia Clinical and Translational Science Awards (CTSAs) as a career navigation tool with a specific focus on CRC career development. The eMPACT^TM^ system evaluates the individual’s skills and competencies and offers direct links to training opportunities in the Translational Workforce Development (TWD) Catalog.

These training opportunities focus on areas necessary for individual growth, institutional protection, and participant safety. HR plays a vital role in onboarding employees and assuring that new hires receive initial training, particularly in the areas of compliance with institutional and regulatory policies. Much less attention has been placed on the training needed for continued professional growth and expansion of skill sets beyond entry-level CRC positions.

Hiring a CRC without the required key skills or abilities, resulted in dissatisfaction by the PI. Respondents highlighted cases where individuals “oversold” their abilities or lacked the necessary knowledge and skills on hire, without clear resources for development of the new hire. This contributed to their dissatisfaction with the CRC, and some respondents expressed that they would not hire the individual again if given the chance. Additionally, some mentioned resorting to a “trial-and-error” approach to complete tasks in the absence of trainings. The eMPACT^TM^ system addresses this problem by guiding CRCs through different levels of training and providing documentation of the skills they have obtained to future employers.

Studies evaluating the CRC workforce reported a significant percentage of CRCs lacking research experience and receiving minimal to no training after hire [15,16]. Owen-Pickle and colleagues found 75% of clinical research associates held a higher education degree (bachelor’s or master’s) but 42% lacked clinical research experience and 74% had little to no training upon hire [13]. The ability to rapidly assess employee training needs on hire and provide accessible, high-quality training opportunities to support the CRC is key to research project operations. A lack of accessible training and career advancement options has been identified as a significant factor contributing to turnover among CRCs [12,13,17,18].

From National Institutes of Health (NIH) CTSAs support, repositories of trainings for clinical research professionals have emerged. However, challenges in accessibility especially content restricted by institutional firewalls, ensuring quality and accuracy, and sustainability have surfaced. Establishing mechanisms to assess training materials that align with best practices, skills, and the competency-based framework essential to the field must be addressed [2,17–20]. The lack of quality trainings limits opportunities for advancement which leads to dissatisfaction with salary, which has been identified as a significant factor contributing to turnover among CRCs [2,12,19]. The grass-roots clinical research professional special interest group within ACTS, which includes members from various CTSA/CTSI groups, has been instrumental in data gathering and publishing on issues related to clinical research professional recruitment, retention, and workforce training needs [20–23].

The availability of flexible and accessible, quality trainings geared toward the adult learner, plays a crucial role in enhancing the skills and competencies of CRCs and advancing professional development [20,21,24]. Institutions that offer onsite trainings, such as lunch and learns and symposia have reported varying attendance to these events, particularly post-COVID. Time constraints and flexible schedules often hinder CRC attendance at these events [20,25]. Addressing these concerns led to the development of the TWD Catalog, a collaborative effort between the Georgia CTSA and the University of Southern California-CTSI. This initiative serves as an open-source repository of trainings for clinical research professionals. Its offerings are approved by an accredited provider, enabling participants to earn certificates and badges, signifying training quality. These online trainings provide valuable resource materials and can be used for meeting certification renewal and annual review requirements, as well as identifying areas for development and career advancement [26,27]. Other resources to facilitate clinical research professional development include the DIAMOND portal, an online educational portal for shared competency-based educational offerings [28,29].

### Engagement with HR

Our study revealed that job descriptions for CRC positions may not always match project needs accurately or the skill set the CRC needs. We are developing initiatives with our eMPACT^TM^ system to engage PIs in understanding the complex skills and competency levels of CRCs and the importance of not just recruiting a person whose base salary matches the funds available on the project to support their salary. Our work with PIs in making their hiring decisions also includes working with our HR department to more effectively identify project and candidate needs.

The onboarding process requires an evaluation of gaps in knowledge and economically viable access for the institution to in-house and external trainings beyond basic organizational requirements. Effective methods of assessing competencies of CRC at the point of hire are needed. We are working with HR to introduce our eMPACT^TM^ system to new hires at the time of onboarding so that the new hire has an inventory of their skills and trainings to promote their job satisfaction and help to avoid a mismatch between the new hires' competencies and the PI expectations.

## Conclusion

The care and safety of clinical research participants are paramount in clinical research, necessitating a well-trained workforce to mitigate risk to participants, PIs, and the research institution. The complexity and evolving nature of the field, coupled with varied levels of experience among professionals, highlights the need for well-written job descriptions that clearly outline roles and responsibilities. Effective onboarding processes that quickly identify training and development needs, along with the availability of professional development tools and activities, are crucial to supporting the growth and success of CRCs. A lack of awareness of specific onboarding needs of new hires emphasizes the value of providing a structured checklist to aid in customizing positions and ensuring better alignment between CRCs and project expectations. Moreover, exploring cost-effective alternatives or funding options can help make external courses and advanced trainings more accessible, promoting continuous professional development and career advancement. The success of CRCs in their roles is vital for the effective conduct of clinical research. By prioritizing well-written job descriptions, targeted onboarding processes, and accessible professional development opportunities, institutions can reduce attrition, improve satisfaction among CRCs and PIs, and ultimately enhance the quality and safety of clinical research studies.
